# Factors associated with child hunger among food insecure households in Bangladesh

**DOI:** 10.1186/s12889-017-4108-z

**Published:** 2017-02-16

**Authors:** Md Ahshanul Haque, Fahmida Dil Farzana, Sabiha Sultana, Mohammad Jyoti Raihan, Ahmed Shafiqur Rahman, Jillian L. Waid, Nuzhat Choudhury, Tahmeed Ahmed

**Affiliations:** 1 Nutrition and Clinical Services Division, International Centre for Diarrhoeal Disease Research, Bangladesh, 68, Shaheed Tajuddin Ahmed Sharani, Mohakhali, Dhaka, Bangladesh; 2 James P Grant School of Public Health, BRAC University, Dhaka, 1212 Bangladesh; 3 Helen Keller International, Dhaka, 1212 Bangladesh

**Keywords:** Child hunger, Food insecurity, Bangladesh, Under 5 children

## Abstract

**Background:**

Hunger is associated with food insecurity at the household level and is considered as a global public health problem with long term adverse consequences on children’s health. This study aims to determine the factors associated with child hunger from a nationally representative sample in Bangladesh among food insecure households.

**Methods:**

Data was derived from the Food Security and Nutritional Surveillance Project; 14,712 children aged 6–59 months belonging to food insecure households contributed to the analysis. Information on food security at the household level was collected for 30 days preceding the survey. Descriptive statistics served to illustrate the variables studied and multiple logistic regression analysis was conducted to identify the significant risk factors for child hunger.

**Results:**

Overall 10% of the children were found to be hungry. After adjusting for seasonality, residence type and education level of household head, the variables - female headed households [OR: 1.87 (1.43–2.45); *p* < 0.001], severely food insecure households [OR: 10.5 (1.43–76.6); *p* < 0.05], households having women with no education [OR: 1.56 (1.27–1.92); *p* < 0.05], poorest asset quintile [OR: 1.50 (1.11–2.15); *p* < 0.05] and the amount of rice consumed per household per week [OR: 0.94 (0.92–0.96); *p* < 0.001] were found to be significantly and independently associated with child hunger.

**Conclusions:**

Out of the potential risk factors examined, our study found significant and independent association of five variables with child hunger: sex of the household head, household food insecurity status, educational status of household women and asset index. Despite all sampled household being food insecure, degree of household food insecurity status appeared to be the strongest predictor of child hunger.

**Electronic supplementary material:**

The online version of this article (doi:10.1186/s12889-017-4108-z) contains supplementary material, which is available to authorized users.

## Background

Food security is a complex development issue which is linked to health and nutrition. Food insecurity is strongly associated with hunger and poverty and is considered as a global public health problem with long term adverse consequences on children’s health [1, 2]. It is a situation which can be described as “limited or uncertain availability of nutritionally adequate and safe foods or limited or uncertain ability to acquire acceptable foods in socially acceptable ways” [3]. Adequate food is defined by the World Food Summit as “all people at all times having access to sufficient, safe, nutritious food to maintain a healthy and active life”[4] and the right to adequate food is a universal human right. However, in situations, when someone cannot acquire adequate amount of food even for a short duration is described as ‘hunger’ [5].

Food insecurity is often rooted in poverty and is of great importance due to its long-term impact on the capacity of families, communities and countries for development [6]. The social concept of hunger which is linked to poverty [7] can be described as a product of food insecurity [8]. Hunger in children pertaining to food insecurity, has been found to be associated with detrimental mental and physical outcomes [9]. Bangladesh is a country in the South Asian region, a region which has a higher growth rate of population compared to other parts of the world and hunger is highly prevalent [1]. Of relevance is that two thirds of all people classified as being ‘hungry’ reside in Asia, with a significant portion chronically lacking access to optimal amount of food [1].

Despite significant economic progress, Bangladesh remains highly food insecure [10–12] with more than 40 million of its people being ‘hungry’ [13]. Bangladesh has been ranked 73^rd^ out of 104 developing and transitioning countries in the recent Global Hunger Index [14]. Hunger is synonymous to undernutrition [15] and at least 14% of all Bangladeshi children under five years of age suffers from some manifestation of undernutrition, with 36% suffering from the chronic form - stunting [11]. Children, focus of this paper, are particularly vulnerable to undernutrition and hunger among all the age groups with one child dying every five seconds from causes related to hunger [16]. Much research has been done in the country to understand issues related to child health and undernutrition, however, little is known about hunger and the factors determining child hunger along with its consequences in the country due to scarcity of pertinent data. A recently concluded large cross-sectional study in Bangladesh, the Food Security Nutritional Surveillance Project (FSNSP), which tracked food security status and nutritional condition throughout Bangladesh, has provided unprecedented opportunity for the assessment of hunger associated with household food insecurity and other relevant contributing factors [17].

Thus the objective of this paper has been set to determine the factors associated with child hunger among food insecure households by analyzing data from a nationally representative sample in Bangladesh collected through the FSNSP.

## Methods

### Study context

**Fig. 1:**
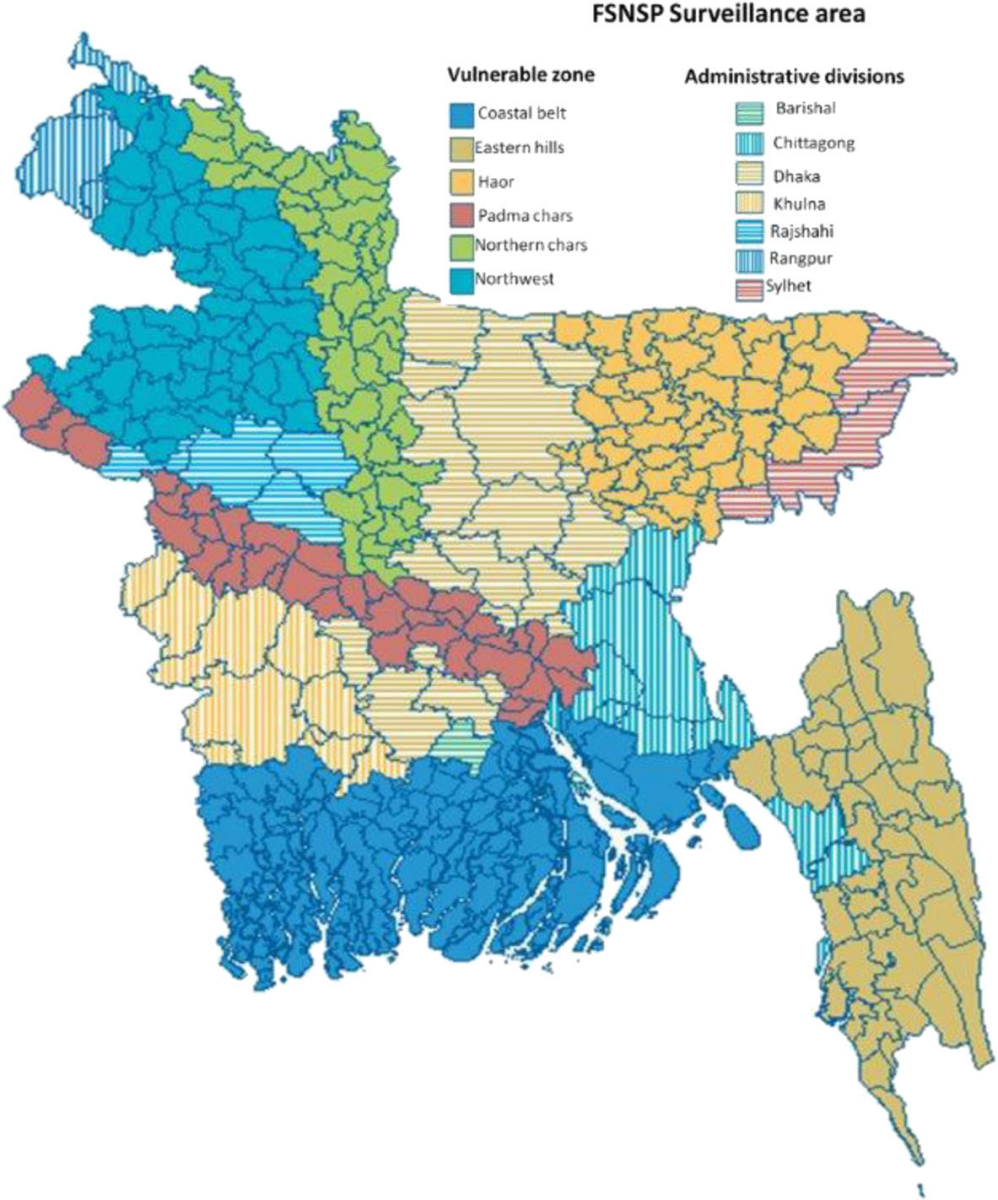
FSNSP surviellance area

**Fig. 2:**
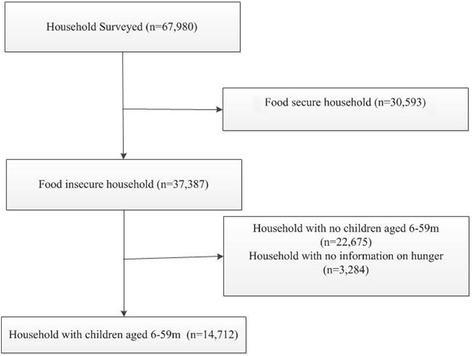
Trial profile

Data was derived from FSNSP, a surveillance system that operated to track nutrition and food security. FSNSP followed a repeated cross-sectional design for collecting data countrywide at the household level. The data was collected after the two major harvest seasons, the *post-aman* harvest season (January-April) and the *post*-*aus* harvest season (September-December) and also during the *monsoon* season (May-August). The study area, the whole country, was divided into 13 strata consisting of six vulnerable zones (coastal belt, eastern hills, haor region, padma chars, northern chars and the northwest region) and the seven administrative divisions (Dhaka, Chittagong, Rajshahi, Barisal, Khulna, Sylhet and Rangpur) which contain all the *upazilas* not included in a surveillance zone, correspond to the seven divisions of Bangladesh. A map of FSNSP zones is illustrated in Fig. 1. From each strata, a set number of *upazilas* were selected with replacement for further sampling based on the number of *upazilas* in the strata. From each of the surveillance zones, *upazilas* were selected by rotation into the sampling frame in order to reduce random variation in estimates between rounds, as has been recommended for surveillance systems by the United Nations (UN) and is commonly done in labour participation surveillance [18]. FSNSP’s rotational pattern ensures that 50% of all *upazilas* in zones are identical between the same season in subsequent years and between two consecutive rounds. In each round, three new *upazilas* were selected for sampling and the remaining nine *upazilas* are drawn from past rounds in each surveillance zone. Each selected upazila remains in the system for four rounds of data collection. In the second stage of sample selection, three rural villages or urban *mohalla* were chosen at random and without replacement from all the villages/*mohalla* in each selected *upazila*. There was no stratification of rural and urban areas during the second stage of selection. The third stage of sample selection was done in the field. In each community, 32 households were selected systematically and interviewed. The starting point for interviews in each village was the first eligible house from a randomly assigned approach road (north, south, east, or west) determined by a random number generator. The next and subsequent households for interview were chosen systematically by counting five or ten households from the previously interviewed household (depending on the size of the village) and, in a “zigzag” fashion, selecting households from both sides of the road. In situations where the identified household was not eligible for inclusion or refused participation, the next household that met the inclusion criteria was selected [19]. Sample size was calculated to obtain representative prevalence estimates for indicators of food insecurity and children’s and women’s undernutrition by surveillance zone. Sample size calculations were based on the estimated prevalence of seven key indicators such as Round-wise estimation of acute childhood malnutrition, child underweight, chronic childhood malnutrition, proportion of women with chronic energy deficiency, proportion of households with food insecurity, proportion of households with “household food deficit”, and proportion of households with poor or borderline food consumption patterns. A trial profile is shown as Fig. 2. The primary respondent was the mother. Data quality was ensured through multiple procedures of review and cross-checking. Monitoring officers reviewed all questionnaires on the day of completion by the data collectors so that any errors or inconsistencies identified could be corrected in the field. Quality control officers revisited a randomly selected sub-sample (around 10%) of interviewed households within 48 h of the initial visit by the data collection team to verify the quality of data collected. For all three rounds conducted in the second field year of FSNSP, internal FSNSP quality control operations were supplemented by Bangladesh Bureau of Statistics (BBS) staff performing a 10% post-enumeration check using a shortened questionnaire. Quality control data were compared to the surveillance data collected by data collectors. Inconsistencies were reviewed by the project manager, project coordinator, training officer, and field managers to identify possible reasons for the discrepancy and to implement appropriate solutions, such as a review session on selected indicators during the refresher training or a revision of the questionnaire.

Data of 14,712 Bangladeshi children aged 6–59 months belonging to food insecure households as per Household Food Insecurity Access Scale (HFIAS) collected between June 2011 and November 2013 was analyzed for this paper. Information on food insecurity at the household level was collected for the month preceding the survey. FSNSP measures food security through HFIAS which defines food insecurity based on lack of access originated due to poverty rather than shortage of supply [20]. The scale contains 9 questions (Worry about food/unable to eat preferred foods/eat just a few kinds of foods/eat foods they really do not want eat/eat a smaller meal/eat fewer meals in a day/no food of any kind in the household/go to sleep hungry/go a whole day and night without eating) to assess the level of anxiety and uncertainty of the participants about household food supply, insufficient quality of food and insufficient food intake [21]. Data has been collected about child hunger separately in this project so as to determine its relation with food insecurity; to ascertain whether child hunger increases with food insecurity.

### Variables of interest

Child hunger was defined in this study as a household having at least one child who consumed only rice or an amount of food less than required or fewer meals than required or went to bed hungry or a whole day and night without food on the day preceding the survey. The household with such a child was defined as a household having at least one hungry child. Cited literature suggested the selection of variables for this study, the selected variables were seasonality, residence type, sex of household head, educational level of household head, educational status of household women, degree of household food insecurity, amount of rice consumed by the household members and asset index. Seasonality, for this paper, has been defined as the segregation of the year into the *post*-*aman* (January-April), *monsoon* (May-August) and *post*-*aus* (September-December) periods, type of residence has been dichotomized into rural and urban. Education level of household head has been categorized into ‘no formal education’, ‘below Secondary School Certificate (SSC)’ and ‘SSC complete and above’, educational status of household women was determined on the basis of households having or not having at least one woman, with at least, one year of formal schooling. The asset index used in this study is a composite indicator of household wealth calculated using principal component analysis, following a method similar to that used in the Bangladesh Demographic and Health Survey (BDHS) [11, 22]. The calculation was based on ownership of household electrical appliances, furniture, livestock and vehicles, the type of household construction materials, kitchen fuel and latrine used and the source of drinking water. Food insecurity was categorized as mildly food insecure, moderately food insecure, and severely food insecure.

### Statistical analysis

All analyses were conducted in STATA v10 (StataCorp; College Station, Texas, USA) using the *svyset* command to adjust strata and cluster for complex survey data. Descriptive statistics served to illustrate the general characteristics and simple logistic regression was used to assess the strength of association (unadjusted) between child hunger and other variables. Multivariate logistic regression analysis was conducted to identify the statistically significant risk factors for child hunger. Logistic regression is the most suitable mode of analysis since the outcome variable is binary and both categorical and continuous variables can be fitted into the regression model. All variables which had *p*-value significant at 0.25 were included in the logistic regression [23]. In the final multiple logistic regression model, variables were considered statistically significant only if the *p*-value was less than 0.05. Seasonality, residence type, educational status of household head were adjusted in all the steps of the model.

## Results

### General characteristics

Our analysis suggests that 14,712 households were food insecure among which 11,428 households had data on hunger and had at least one child aged 6–59 months. Around 94% households were from rural area. The average number of household member was five and the average consumption of rice by each household was 13 kg in the week preceding the survey. Among households who experienced hunger, 81% were severely food insecure. Overall, 10% of the children were hungry and additionally, around 1% went a whole day and night (24 h) without eating anything or slept at night being hungry. On the basis of seasonality, the proportion of hungry children was around 8% during *post*-*aman*, 10% during *monsoon* and 10% during *post*-*aus* season. Ninety three percent of the households were headed by male and 51% of the household heads had no formal education. Around 59% of the primary earner of the households was day laborer, whereas around 16% households had no women with at least one-year of formal education. All the descriptive findings are tabulated in Table 1.

**Table 1:** General characteristics of the subjects

Variables (N)		n (%)
Amount of rice consumed (9369) mean (SD)		12.93 (6.37)
Child hunger (11428)		
	Overall	1086 (9.51)
	Severe hunger	109 (0.95)
Seasonality of child hunger		
	Post-*aman*	210 (8.40)
	Monsoon	428 (9.50)
	Post-*aus*	448 (10.13)
Place of residence (14712)		
	Rural	13857 (94.19)
	Urban	855 (5.81)
Sex of the household head (14712)		
	Male	13641 (92.72)
	Female	1071 (7.28)
Education level of household head (14684)		
	No formal education	7539 (51.34)
	Below secondary school certificate	6360 (43.31)
	Secondary school certificate complete and above	785 (5.35)
Occupation of primary earner of the household (9369)		
	Farmer	1508 (16.10)
	Day laborer	5558 (59.32)
	Businessman	1233 (13.16)
	Professional	769 (8.21)
	Others	301 (3.21)
Household food insecurity status (14712)		
	Mild food insecurity	1632 (11.09)
	Moderate food insecurity	1172 (7.97)
	Severe food insecurity	11908 (80.94)
Women education status (14712)		
	No educated women in the household	2356 (16.01)
	At least one educated women in the household	12356 (83.99)
Asset index (14712)		
	1^st^ quintile	3772 (25.64)
	2^nd^ quintile	3874 (26.33)
	3^rd^ quintile	3226 (21.93)
	4^th^ quintile	2145 (14.58)
	5^th^ quintile	1695 (11.52)

### Strength of association

Our bivariate and multivariate analyses (Table 2) revealed that seasonality, type of residence as well as education level of household head was not significantly associated with child hunger. The odds of child hunger was 1.87 times [95% CI: 1.43–2.45; *p* < 0.001] in female headed households compared to male headed households. Severe food insecurity in the households appeared to be significantly associated with child hunger [OR: 10.5 (1.43–76.6); *p* < 0.05] in comparison to mildly food insecure households. It was also found that child hunger was 1.56 times [95% CI: 1.27–1.92; *p* < 0.05] more in a household having no educated woman. Odds of a child being hungry in the poorest household was 1.54 times higher [95% CI: 1.11–2.15; *p* < 0.05] compared to the richest quintile. The amount of rice consumed by the household members in the past week was negatively associated with child hunger [OR: 0.94 (0.92–0.96); *p* < 0.001].

**Table 2:** Determinants of child hunger

Variables		*n*	Unadjusted OR (95%CI)	*p*-value	Adjusted OR (95%CI)	*p*-value
Seasonality		11428				
	Post-*aus*		Reference		Reference	
	Post-*aman*		0.81 (0.64–1.03)	0.080	1.06 (0.82–1.38)	0.638
	Monsoon		0.93 (0.77–1.12)	0.448	1.12 (0.89–1.40)	0.333
Residence type		11428				
	Urban		Reference		Reference	
	Rural		0.99 (0.73–1.35)	0.976	1.23 (0.86–1.75)	0.256
Sex of the household head		11428				
	Male		Reference		Reference	
	Female		1.84 (1.52–2.23)	0.000	1.87 (1.43–2.45)	0.000
Education level of household head		11410				
	Secondary school certificate complete and above		Reference		Reference	
	No formal education		1.39 (0.94–2.05)	0.097	0.91 (0.55–1.49)	0.702
	Below secondary school certificate		1.28 (0.86–1.89)	0.220	0.93 (0.57–1.53)	0.784
Occupation of primary earner of the household		7290				
	Businessman		Reference		Reference	
	Farmer		0.98 (0.69–1.40)	0.921	0.99 (0.70–1.41)	0.985
	Day laborer		1.41 (1.10–1.81)	0.007	1.22 (0.95–1.56)	0.122
	Professional		1.08 (0.71–1.65)	0.722	0.97 (0.62–1.51)	0.876
	Others		2.26 (1.44–3.55)	0.000	1.29 (0.80–2.09)	0.293
Household food insecurity access scale		11428				
	Mildly food insecure		Reference		Reference	
	Moderately food insecure		0.96 (0.43–2.14)	0.924	5.07 (0.67–38.6)	0.116
	Severely food insecure		2.19 (1.06–4.49)	0.033	10.5 (1.43–76.6)	0.021
Women education status		11428				
	At least one educated women in the household		Reference		Reference	
	No educated women in the household		1.60 (1.36–1.88)	0.000	1.56 (1.27–1.92)	0.000
Asset index		11428				
	5^th^ quintile		Reference		Reference	
	1^st^ quintile		2.09 (1.54–2.85)	0.000	1.54 (1.11–2.15)	0.011
	2^nd^ quintile		1.79 (1.33–2.41)	0.000	1.31 (0.94–1.83)	0.111
	3^rd^ quintile		1.44 (1.05–1.98)	0.024	1.09 (0.76–1.57)	0.633
	4^th^ quintile		1.08 (0.76–1.54)	0.653	0.91 (0.59–1.40)	0.654
Amount of rice consumed		7290	0.93 (0.91–0.95)	0.000	0.94 (0.92–0.96)	0.000

## Discussion

Data used in this paper was collected through FSNSP that followed a cross-sectional design with the objective to determine factors which potentially contributes to child hunger among food insecure households. Nearly one tenth children of the current study were found to be hungry. Factors which determined child hunger identified by our study were female household head, severe food insecurity, women with no education, poorest asset quintile and amount of rice consumed per week.

Our multivariate analysis showed that, the odds of child hunger was significantly higher among severely food insecure households which is supported by similar studies [24, 25]. In the FSNSP study, more than three fourth of the households were found to be severely food insecure.

It should also be noted that a significant proportion of the Bangladeshi population remains food insecure despite considerable economic development. Dependency on manual labor and use of traditional techniques with locally available tools in agriculture largely affect crop production and influences food availability; as a result, prevalence of food insecurity is high [12, 26].

One of the significant findings of this study, was that female headed households had nearly two-fold risk of having a hungry child. Many factors may contribute to this vulnerability including lower earnings, limited access to assets, land and property and lack of social protection. Women were also more likely to be deprived in many other important areas of well-being, such as education, which our study has also found to be correlated with child hunger. In a study done in Southern Ethiopia, increased maternal education was also found to be associated with lower food insecurity and hunger [27]. It is likely that educated females are better equipped, be financially independent, have more control or influence on household resource allocation which lead to a lowering of financial and ultimately, of food insecurity and also ensures better nutrition for children [28, 29]. Educated women additionally, may be, more skillful in domestic financial management. Evidence also suggests that educated mothers are more capable of coping with the many unwritten restrictions and obstacles present in a male dominated society [30]. Factors pertaining to improved child nutrition such as birth spacing and having fewer children are also associated with the education status of the mother [31, 32]. In line with our study, established association between household asset index and hunger is seen in published literature [33–35]. The poorest segments of society are in general often most vulnerable to serious economic crisis leading to worsening food and nutrition security at the household level [36]. The world’s extremely poor are distributed unevenly across regions and countries. The majority of people living on less than $1.25 a day reside in two regions—Southern Asia and sub-Saharan Africa accounting for about 80% of the extremely poor globally. According to an estimation made in 2011, about 60% of the world’s extremely poor people lives in just five countries, one of which is Bangladesh [37]. All of these factors may contribute towards greater stability and security regarding food.

Economic growth is necessary, but not sufficient for sustaining progress made in the reduction of poverty and hunger. Approximately three-quarters of the world’s poor live in rural areas, making up a high percentage of the hungry and malnourished in developing countries [38, 39]. The same is true for Bangladesh. Thus inclusive growth which enables rural poor to diversify livelihood, is critical to reduce hunger. Lack of purchasing power and ultimately, the lack of access to food, especially for the rural ultra-poor people compel them to remain food insecure. Additionally, adequate food availability at the household level does not necessarily imply that all members of a household enjoy access to enough food. In particular, women and children often suffer from inequalities in intra-household food distribution [40].

Efforts to promote growth in agriculture and the rural sector can be an important component for promoting food security [38]. Another strategy that may be considered is fostering social protection systems efficiently as it directly contributes to the reduction of poverty, hunger and undernutrition by promoting income especially among women [39]. Lastly, as our findings dictate, since educational status of household women is significantly associated with child hunger, emphasis should be given towards women education. The results of this study confirms the significant contribution of relevant socio-demographic and other characteristics towards child hunger among food insecure households.

### Limitations and strength

While the explanatory variables indicate risk factors for child hunger, causal inferences cannot be established due to the cross-sectional nature of the data. A possibility of recall bias remains regarding HFIAS data, as information of 1 month preceding the survey was gathered through maternal response. While, the strength lies in the large sample size and adjustment of seasonality along with separate measurements for food insecurity and hunger. Nevertheless, the present work contributes greatly to our understanding of the socio-economic characteristics related to child hunger among food insecure households.

## Conclusions

As conclusive remarks, it could be said that out of the potential risk factors examined, our study found significant and independent association of five variables with child hunger: sex of household head, primary earner of household, household food insecurity status, educational status of household women and asset index. Degree of household food insecurity status appeared to be the strongest predictor for child hunger among food insecure households. Infants and young children belonging to severe food insecure households are especially at higher risk of being hungry. Efforts directed towards achieving food security are unlikely to be successful if the issue of child hunger is ignored. This paper has tried to shed light on the factors at the food insecure households that compel all member including children to face hunger. This can serve as a result base on which further studies can be conducted to gain more in-depth information about child hunger and relevant areas.

## Additional file

Additional file 1: Food Security and Nutrition Surveillance Data. This data set is a minimal one from a large data set of surveillance on food security and nutrition. It contains unique identification number, region, seasonality, sociodemographic characteristics, food security status and information on child hunger. (DTA 2080 kb)

## Abbreviations

BDHS: Bangladesh Demographic and Health Survey
BU: BRAC University
EU: European Union
FSNSP: Food Security Nutritional Surveillance Project
HFIAS: Household Food Insecurity Access Scale
icddr,b: International Centre for Diarrhoeal Disease Research, Bangladesh
